# The Ramathibodi early warning score as a sepsis screening tool does not reduce the timing of antibiotic administration

**DOI:** 10.1186/s12245-022-00420-w

**Published:** 2022-05-10

**Authors:** Karn Suttapanit, Kamonwan Dangprasert, Pitsucha Sanguanwit, Praphaphorn Supatanakij

**Affiliations:** Department of Emergency Medicine, Faculty of Medicine Ramathibodi Hospital, Mahidol University, Bangkok, Thailand

**Keywords:** Time to antibiotic administration, Trigger tools, Early warning score, Sepsis

## Abstract

**Background:**

Administration of antibiotics to septic patients within 1 h was recommended in 2018 by the Surviving Sepsis Campaign (SSC) as a strategy to improve survival outcomes. The use of sepsis screening tools in emergency departments (EDs) is important for early diagnosis and initiation of sepsis care. This study aimed to assess the impact of the Ramathibodi early warning score (REWs) on the administration of antibiotics within 1 h of presentation.

**Methods:**

This was an observational retrospective cohort study with propensity score matching between the sepsis-3 criteria (pre-period) and the REWs (post-period) as screening tools in adult patients with sepsis in EDs. The primary outcome was the proportion of receiving antibiotics within 1 h of presentation in the pre- and post-periods.

**Results:**

A total of 476 patients were analyzed without propensity matching. The proportion of antibiotic administration within 1 h was higher in patients screened using the REWs compared with standard of care in the total study population (79.5% vs. 61.4%, *p* < 0.001). After propensity score matching, 153 patients were included in both groups. The proportion of antibiotic administration within 1 h was similar in patients screened using the REWs and those receiving standard of care (79.7% vs. 80.4%, *p* = 0.886). However, time to intensive care unit (ICU) admission was faster in patients screened using the REWs. Delays in receiving antibiotics of longer than 3 h were associated with increased mortality (adjusted hazard ratio 7.04, 95% confidence interval 1.45 to 34.11, *p* = 0.015).

**Conclusions:**

Implementing the REWs as a tool in sepsis screening protocols in EDs did not improve rates of antibiotic administration within 1 h as recommended by the SSC. However, time to ICU admission was improved after implementation of the REWs.

**Supplementary Information:**

The online version contains supplementary material available at 10.1186/s12245-022-00420-w.

## Background

Sepsis is a major cause of mortality associated with emergency department (ED) visits [1–3]. In 2018, the Surviving Sepsis Campaign (SSC) recommended the following strategy to improve patient survival rates: (i) blood cultures and blood lactate measurements should be performed immediately, (ii) empirical antibiotics should be administered within 1 h of recognition of the signs of sepsis, and (iii) adequate fluid resuscitation should be given, and vasopressor use should be implemented in patients who remain hypotensive after fluid resuscitation [4].

Screening for sepsis in EDs is important for early diagnosis and initiation of sepsis care [1, 5]. In previous studies, several sepsis screening tools (e.g., the systemic inflammatory response syndrome, the early warning score, the quick Sequential Organ Failure Assessment (qSOFA), and lactate measurements plus qSOFA) on mortality rates were compared [6–8]. However, the screening tool’s impact on the sepsis bundle was remained unclear. Several studies show administering appropriate antibiotics within 3 h in patients with suspected sepsis and within 1 h in patients with septic shock was associated with increased survival rates [9–11]. However, few studies have assessed the impact of screening tools in improving antibiotic administration and ICU admission time. Sepsis patients with delayed ICU admission from the ED were related to increased mortality and adverse outcomes [12, 13]. Delayed ICU admission of more than 6 h had increased mortality in critically ill patients [14]. The recent data shows a sepsis alert system was established to enable early identification and initiation of therapy and decreases the length of stay in the ED in a patient with sepsis [15–17].

This study assessed the impact of the Ramathibodi early warning score (REWs) on the administration of antibiotics within 1 h of presentation, time to intensive care unit (ICU) admission, frequency of ICU admission, and 28-day mortality in patients with sepsis.

## Methods

### Study design

This observational retrospective cohort study with propensity score matching was conducted at Ramathibodi Hospital, a tertiary care and university hospital in Bangkok, Thailand. The study protocol was approved by the Ethics Committee of Ramathibodi Hospital, Mahidol University (COA MURA2020/505). The ethics committee waived the need for informed consent of each patient.

### Study setting and population

The study enrolled patients 18 years and older who visited the Ramathibodi Hospital ED with suspected sepsis and were screened using the Ramathibodi sepsis protocol from August 1, 2019, to December 31, 2019. The exclusion criteria were as follows: (i) patient and/or family submission of a do-not-attempt resuscitation order, (ii) transfer to another hospital, (iii) treatment at another hospital before arrival at our ED, and (iv) missing vital signs, time of antibiotic administration, time of ICU admission, or mortality.

In Ramathibodi Hospital, during the pre-period (August 1, 2019, to October 29, 2019), patients with suspected sepsis were managed according to the standard of care defined by the sepsis-3 criteria. At the triage area, the nurse triggered a sepsis alert system in patients with an initial qSOFA score ≥ 2 or suspected sepsis and tracked patients to physicians for judgment following sepsis protocols. The REWs was provided in the ED on October 30, 2019, due to the sepsis committee of Ramathibodi Hospital. During the post-period (October 30, 2019, to December 31, 2019), patients were tracked following sepsis protocols, including REWs screening. If the REWs ≥ 4 points, the triage nurse alerted sepsis protocol, and patients were managed following the same sepsis protocols as those used during the pre-period. In addition, during the post-period, REWs were implemented as part of the continuity of care process, including monitoring and notifying physicians to evaluate patients.

The emergency physicians and nursing teams, the number of medical personnel per shift in the ED, and the process for administration of antibiotics were the same during the pre- and post-periods. All ED physicians and residents were trained in the use of sepsis protocols and in management of patients with sepsis.

### Definitions

Suspected sepsis was defined by qSOFA score ≥ 2 as a sepsis-3 criteria or physician judgment suspected infection in pre-period and REWs ≥ 4 in post-period that consists of lactate measurement, taking hemoculture and antibiotic administration. The REWs score was defined based on clinical parameters including systolic blood pressure, heart rate, respiratory rate, pulse oximetric saturation, body temperature, and mental status. Each parameter was scored as shown in Supplementary Online Table 1.

### Measures

Patients included in this study were evaluated by emergency room staff. We recorded each patient’s demographic information (age, sex, and comorbidities), vital signs (systolic blood pressure, respiratory rate, body temperature, and pulse oxygen saturation), qSOFA score, and REWs in the triage area. Blood lactate levels were assessed upon admission. Data collected from hospital database software were used for this study. Additionally, the medical record was audited by an internal auditor in Ramathibodi Hospital, which uses medical record audit guideline 2021 by the Healthcare Accreditation Institute of Thailand (public organization).

The primary outcome of this study was the impact of the REWs on the administration of antibiotics within 1 h of presentation. The secondary outcomes were time from presentation to ICU admission and 28-day mortality rate among patients with sepsis treated according to standard of care (sepsis-3) and after addition of the REWs trigger tools to sepsis protocols.

### Data analysis

The sample size required for this study was calculated based on a pilot study in the in-patient department. This study found that the proportion of patients with suspected sepsis who received antibiotics within 1 h as part of standard of care (sepsis-3) was 38%, and that following implementation of the REWs, the mean early warning score was 0.57. Therefore, this study required a sample size of 150 patients in each group assuming an alpha error of 0.05 (two-sided) and a power of 0.9.

Categorical variables were expressed as counts and percentages, and continuous variables were expressed as medians and interquartile ranges (IQRs). A propensity score (PS) generated from a multivariate logistic regression model was calculated for trigger tools. A total of 21 variables were used to generate the PS. The PS from the regression model (with nearest neighbor 1:1 matching without replacement) was derived with a caliper of 0.05.

For analysis of the pre-and post-propensity score matching groups, categorical variables were compared using Pearson’s chi-square test, and continuous variables were compared using Mann-Whitney *U*-tests. Multivariate Cox regression was used to assess associations between time to antibiotic administration and 28-day mortality.

Statistical analyses were performed using IBM SPSS Statistics for Windows, version 22.0 (Armonk, NY, USA), and STATA version 16.1 (StataCorp, College Station, TX, USA).

## Results

**Fig. 1:**
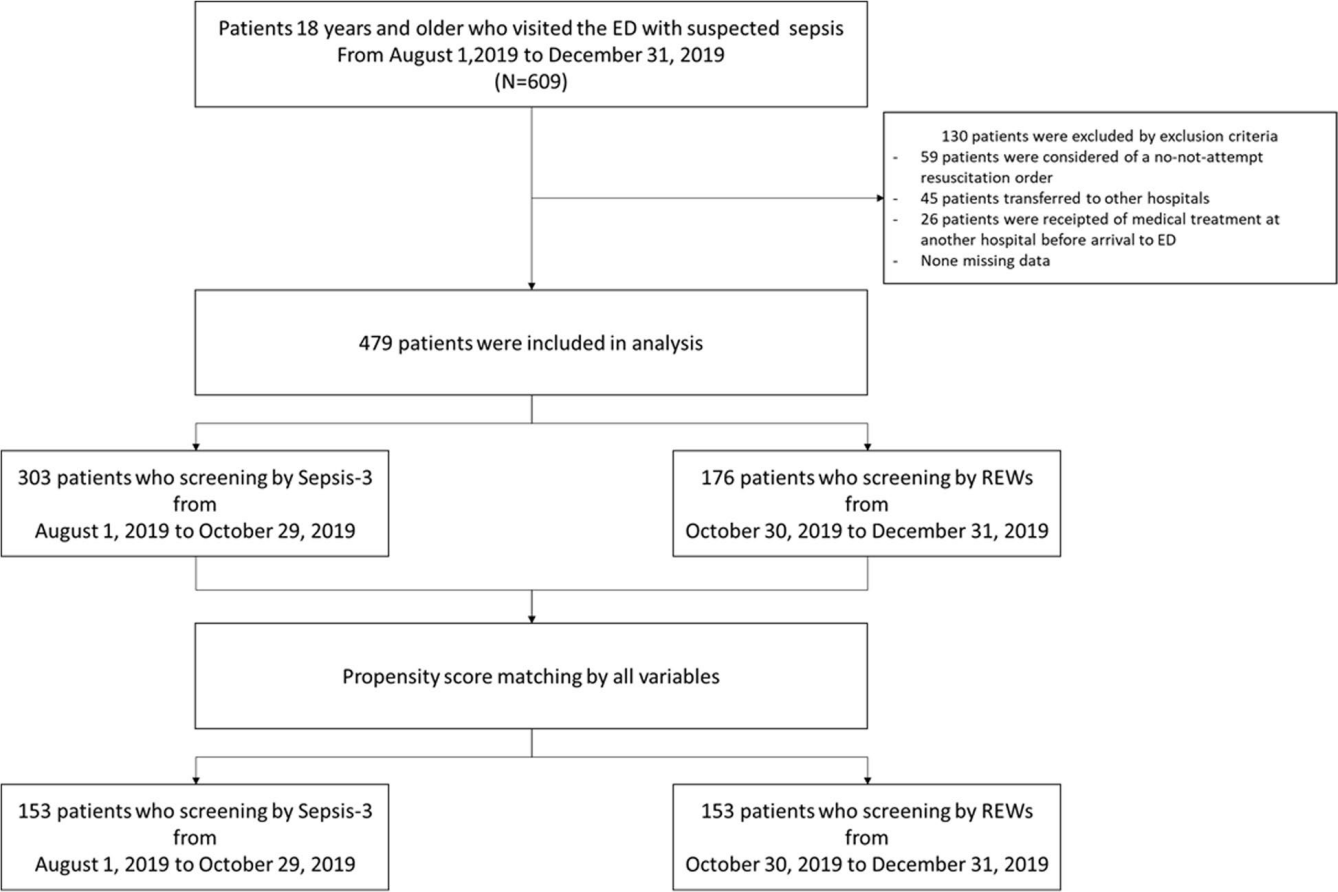
Study flow

A total of 609 patients were screened, and 130 patients were excluded according to the eligibility criteria, leaving 479 patients in the final analysis. Among these 479 patients, 303 patients were evaluated using the sepsis-3 criteria, and 176 were evaluated using the REW. After matching by propensity scores, 153 patients remained in both groups as shown in Fig. 1. All baseline variables were similar between the propensity score-matched populations. Demographic information, physiological characteristics, site of infection, qSOFA scores, REWs, and blood lactate levels in the pre-and post-propensity score matching populations are shown in Table 1.

**Table 1:** Baseline characteristics of patients screened using sepsis-3 and REWs with and without propensity matching

Characteristics	Total cohort			Propensity matching		
	Sepsis-3 (***N*** = 303)	REWs (***N*** = 176)	***p***-value	Sepsis-3 (***N*** = 153)	REWs (***N*** = 153)	***p***-value
**Male gender,*N*(%)**	135 (44.6%)	72 (40.9%)	0.325	71 (46.4%)	62 (40.5%)	0.299
**Age (years), median (IQR)**	72 (71–75)	71 (68–75)	0.962	71 (68–75)	71 (67–76)	0.693
**Temperature (°C), median (IQR)**	38.3 (38.2–38.6)	38.7 (38.5–38.8)	0.024	38.7 (38.6–39.0)	38.7 (38.6–38.9)	0.943
**Heart rate (beats/min), median (IQR)**	106 (103–108)	116 (113–120)	< 0.001	112 (110–117)	116 (113–120)	0.357
**Respiratory rate (breaths/min), median (IQR)**	22 (22–24)	24 (24–28)	0.001	24 (24–26)	24 (24–28)	0.192
**SBP (mmHg), median (IQR)**	130 (126–135)	129 (122–133)	0.177	124 (118–135)	129 (122–133)	0.881
**SpO**_**2**_**(%), median (IQR)**	96 (96–97)	96 (95–97)	0.064	95 (94–96)	96 (95–97)	0.254
**Altered mental status,*N*(%)**	51 (16.8%)	47 (26.7%)	0.010	32 (20.9%)	36 (23.5%)	0.582
**Comorbidities,*N*(%)**						
**Cirrhosis**	17 (5.6%)	4 (2.3%)	0.085	4 (2.5%)	4 (2.5%)	1.000
**Diabetes mellitus**	122 (40.3%)	53 (30.1%)	0.026	54 (35.3%)	51 (33.3%)	0.718
**Hematological malignancy**	19 (6.3%)	8 (4.5%)	0.430	8 (5.2%)	8 (5.2%)	1.000
**Non-hematological malignancy**	55 (18.2%)	37 (21%)	0.442	27 (17.6%)	32 (20.9%)	0.469
**Transplantation**	10 (3.3%)	5 (2.8%)	0.781	5 (3.3%)	4 (2.6%)	0.735
**Immunocompromised**	52 (18.2%)	37 (21%)	0.295	27 (17.6%)	32 (20.9%)	0.469
**ESRD on RRT**	24 (7.9%)	13 (7.4%)	0.833	8 (5.2%)	11 (7.2%)	0.477
**HFrEF**	9 (3.0%)	6 (3.4%)	0.790	5 (3.3%)	4 (2.6%)	0.735
**COPD**	44 (14.5%)	18 (10.2%)	0.177	22 (14.4%)	16 (10.5%)	0.298
**Totally dependent for ADL**	50 (16.5%)	46 (26.1%)	0.011	34 (22.2%)	36 (23.5%)	0.785
**Site of infection**						
**Respiratory system**	120 (39.6%)	73 (41.5%)	0.687	58 (37.9%)	65 (42.5%)	0.414
**Urinary tract**	76 (25.1%)	36 (20.5%)	0.249	37 (24.2%)	32 (20.9%)	0.494
**Gastrointestinal tract**	43 (14.2%)	25 (14.2%)	0.997	22 (14.4%)	21 (13.7%)	0.869
**CNS**	15 (5.0%)	6 (3.4%)	0.427	6 (3.9%)	5 (3.3%)	0.759
**Skin**	12 (4.0%)	9 (5.1%)	0.552	5 (3.3%)	7 (4.6%)	0.556
**CRBSI**	3 (1.0%)	2 (1.1%)	0.879	3 (2.0%)	1 (0.7%)	0.314
**Other**	34 (11.2%)	25 (14.2%)	0.338	22 (14.4%)	22 (14.4%)	1.000
**Lactate (mmol/L), IQR**	2.10 (2.00–2.30)	2.35 (2.20–2.60)	0.001	2.20 (2.10–2.50)	2.30 (2.20–2.70)	0.342
**qSOFA (IQR)**	1 (1–2)	1 (1–2)	< 0.001	1 (1–2)	1 (1–2)	0.735
**REWs (IQR)**	4 (4–5)	5 (5–6)	< 0.001	5 (5–6)	5 (5–6)	0.730

*REWs* Ramathibodi early warning score, *IQR* Interquartile range, *RR* Respiratory rate, *SBP* Systolic blood pressure, *SpO*_*2*_ Pulse oximetric saturation, *ESRD on RRT* End-stage renal disease on renal replacement therapy, *HFrEF* Heart failure with reduced ejection fraction (left ventricular ejection fraction ≤ 40%), *COPD* Chronic obstructive pulmonary disease, *ADL* Activities of daily living, *CNS* Central nervous system, *CRBSI* Catheter-related bloodstream infection, *qSOFA* Quick Sequential Organ Failure Assessment

After introducing the REWs as the screening tool to trigger sepsis alert, the median time from presentation to antibiotic administration was 45 min (*IQR*, 41 to 50 min). This time from presentation to antibiotic administration was not statistically different from that using sepsis-3 criteria. The proportion of antibiotic administration within 1 h in the REWs group was higher than that in patients receiving standard of care (79.5% vs. 61.4%, *p*-value < 0.001). Moreover, time to ICU admission was significantly shorter in the REWs group than in patients receiving standard of care [11 h (*IQR*, 7–14 h) vs. 20 h (*IQR*, 14–15 h)], *p*-value 0.007). However, a 28-day mortality was not significantly different in patients screened using the REWs and those receiving standard of care (Table 2). In the total cohort, delays in antibiotic administration of longer than 3 h increased the risk of 28-day mortality (Table 3).

**Table 2:** Time management in sepsis bundles and 28-day mortality using sepsis-3 and REWs

**Outcomes**	**Sepsis-3 (*N*= 303)**	**REWs (*N*= 176)**	***p*-value**
**Primary outcomes**			
**Administration of antibiotics within 1 h,*N*(%)**	186 (61.4%)	140 (79.5%)	< 0.001
**Time from presentation to administration of antibiotics (minutes), median (IQR)**	50 (46–56)	45 (41–50)	0.112
**Secondary outcomes**			
**ICU admission,*N*(%)**	45 (14.8%)	42 (23.9%)	0.014
**Time to ICU admission (hours), median (IQR)**	20 (14–15)	11 (7–14)	0.007
**28-day mortality,*N*(%)**	26 (8.6%)	12 (6.8%)	0.491
**Propensity matching**	**Sepsis-3 (*N*= 153)**	**REWs (*N*= 153)**	***p*-value**
**Primary outcome**			
**Administration of antibiotics within 1 h,*N*(%)**	123 (80.4%)	122 (79.7%)	0.886
**Time from presentation to administration of antibiotics (minutes), median (IQR)**	35 (33–38)	45 (41–52)	< 0.001
**Secondary outcomes**			
**ICU admission,*N*(%)**	28 (18.3%)	34 (22.2%)	0.393
**Time to ICU admission (hours), median (IQR)**	23 (16–32)	10 (6–14)	0.006
**28-day mortality,*N*(%)**	14 (9.2%)	10 (6.5%)	0.671

*REWs* Ramathibodi early warning score, *IQR* Interquartile range, *ICU* Intensive care unit

**Table 3:** Impact of time of antibiotic administration on 28-day mortality in patients with sepsis

Time to antibiotic administration	HR (95% ***CI***)	***P***-value	Probability	aHR^**a**^ (95% ***CI***)	***P***-value
< 1 h	Reference		7.36	Reference	
1 to 3 h	1.28 (0.55–3.00)	0.571	7.64	1.95 (0.86–4.57)	0.115
> 3 h	6.41 (1.86–22.13)	0.003	33.33	7.04 (1.45–34.11)	0.015

*HR* Hazard ratio, *CI* Confidence interval, *aHR* Adjusted hazard ratio

^a^Adjusted for blood lactate level ≥ 2 mmol/L, systolic blood pressure < 90 mmHg, SpO_2_ < 94%, mental status change, and ICU admission

Following propensity score matching (Table 2), the proportion of antibiotic administration within 1 h did not differ between patients screened using the REWs and those receiving standard of care (79.7% vs. 80.4%, *p*-value 0.886). However, time to ICU admission was significantly shorter in patients screened using the REWs than those receiving standard of care [10 h (*IQR*, 6–14 h) vs. 23 h (*IQR*, 16–32 h)], *p*-value 0.006).

## Discussion

Implementation of the REWs as a screening tool in sepsis protocols improved the proportion of antibiotic administration within 1 h in the total study population. However, following propensity score matching, the proportion of antibiotic administration within 1 h was not different between patients screened using REWs and those receiving standard of care. Implementation of the REWs reduced time to ICU admission in the pre-and post-propensity matching cohorts but had no apparent impact on 28-day mortality. Delays in antibiotic administration of longer than 3 h were associated with 28-day mortality.

As of 2018, the SSC recommends administering empirical antibiotics within 1 h of recognizing the signs of sepsis [4]. In previous studies, delays in antibiotic administration were associated with increased mortality in patients with sepsis. Ko et al. and Seymour et al. showed that each hour delay in antibiotic administration was associated with increased in-hospital mortality [18, 19]. The SSC 2021 recommends administering antibiotics within 3 h for adults with possible sepsis [20]. Our study confirms this suggestion as delays in antibiotic administration of longer than 3 h increased the probability of 28-day mortality.

Previous studies showed that implementing sepsis alerts in EDs improved compliance with the SSC 2018 bundle and improved survival [21, 22]. Song et al. utilized only the sepsis-3 criteria as a screening tool both pre-and post-implementation of sepsis alerts. The qSOFA criteria had poor accuracy and low sensitivity for predicting mortality in patients with sepsis, as sepsis was not recognized in most patients and the protocol was not activated [6, 7, 23, 24]. Early warning signs had improved accuracy compared with qSOFA in predicting mortality [25, 26]. Few studies have assessed the impact of screening tools in decreasing the time from presentation to antibiotic administration [27]. This study showed that implementation of the REWs as a component of sepsis alerts improved the proportion of antibiotic administration within 1 h in the total study population. However, following propensity score matching, there was no difference in the proportion of antibiotic administration within 1 h in patients screened using the REWs and those receiving standard of care. In addition, there were no differences in the baseline characteristics and clinical parameters between propensity score-matched patients, and the same process was used to track patients with sepsis by triage nurses and emergency physicians.

Early warning score systems are important for prioritizing patients who require a high level of care for transfer to ICUs [28]. Sawyer et al. showed that a real-time alert system reduced time to ICU admission and in-hospital mortality among patients with sepsis [15]. Similarly, our study showed reduced time to ICU admission following the REWs as a screening tool during triage for the continuous care process. However, no improved survival outcomes were observed within 28 days following the REWs. Development of screening tools and alert systems requires time for implementation and to assess the efficacy of tools in improving survival outcomes [20, 27].

### Limitations

This study had several limitations. First, this was a retrospective study of a pre-and post-period. Thus, selection bias may have influenced patients’ baseline characteristics and disease severity in the pre-and post-periods. However, we used propensity score matching to control for confounders between groups. Second, the duration of data collection was 3 months for the standard of care and 2 months following implementation of the REWs. Thus, the impact of experience in using trigger tools in the standard of care was more significant during the post-period, and the Hawthorne effect may have influenced the study results in the post-period. Finally, our study was conducted at a single center, and thus, generalizability of the results to other populations is uncertain. Additional multicenter studies of the REWs are needed to validate its benefits for patients with sepsis.

## Conclusions

After implementing the REWs as a trigger tool in sepsis protocols in EDs, the proportion of antibiotic administration within 1 h was improved in the total cohort. However, following propensity score matching, the compliance of antibiotic administration within 1 h was similar in patients screened using the REWs and those receiving standard of care. Time to ICU admission was shorter for in patients screened using the REWs than with those receiving standard of care; however, no difference in 28-day mortality was observed between patients screened using the REWs and those receiving standard of care. Delays in antibiotic administration of longer than 3 h were associated with increased mortality.

## Supplementary Information

**Additional file 1: Supplement Online Table 1.** Ramathibodi early warning score (REWs) clinical parameters and rubric scale for each parameter.

## Data Availability

The datasets analyzed in this study are not publicly available owing to privacy issues but are available from the corresponding author upon reasonable request.

## Abbreviations

ADL: Activities of daily living
aHR: Adjusted hazard ratio
CI: Confidence interval
CNS: Central nervous system
COPD: Chronic obstructive pulmonary disease
CRBSI: Catheter-related blood stream infection
EDs: Emergency departments
ESRD on RRT: End-stage renal disease on renal replacement therapy
HFrEF: Heart failure with reduced ejection fraction (left ventricular ejection fraction ≤ 40%)
HR: Hazard ratio
ICU: Intensive care unit
IQRs: Interquartile ranges
PS: Propensity score
qSOFA: quick Sequential Organ Failure Assessment
REWs: Ramathibodi early warning score
RR: Respiratory rate
SBP: Systolic blood pressure
SpO_2_: Pulse oximetric saturation
SSC: Surviving Sepsis Campaign
