# Supplementary material for: The Ramathibodi early warning score as a sepsis screening tool does not reduce the timing of antibiotic administration
Source: Int J Emerg Med. 2022 May 10;15:18. doi: 10.1186/s12245-022-00420-w (PMC9087922; doi:10.1186/s12245-022-00420-w)
Supplement: Supplementary file 1 — Additional file 1: Supplement Online Table 1. Ramathibodi early warning score (REWs) clinical parameters and rubric scale for each parameter. [file 12245_2022_420_MOESM1_ESM.docx]

**Supplement Online Table 1.** Ramathibodi early warning score (REWs) clinical parameters and rubric scale for each parameter.

| REWS | | | | | | |
| --- | --- | --- | --- | --- | --- | --- |
| Score | RR | SpO2 | Temperature | SBP | HR | Mental status |
| 3 | <10 | <84 | <33.9 | <89 | <39 |  |
| 2 |  | 85-89 | 34-34.9 |  |  |  |
| 1 |  | 90-92 | 35-35.9 | 90-99 | 40-49 |  |
| 0 | 11-20 | >93 | 36-37.9 | 100-199 | 50-99 | Alert |
| 1 | 21-30 |  | 38-38.9 |  | 100-109 | Response to voice |
| 2 | 31-35 |  | >39.0 | >200 | 110-129 | Response to pain |
| 3 | >36 |  |  |  | >130 | Unresponsive |
